# Treatment of Peripheral Arterial Occlusive Disease around the Globe: Malta

**DOI:** 10.3390/jcm10245747

**Published:** 2021-12-08

**Authors:** Anthony Pio Dimech, Samuel Anthony Galea, Kevin Cassar, Matthew Joe Grima

**Affiliations:** 1 Department of General Surgery, Vascular Unit, Mater Dei Hospital, MSD 2090 L-iMsida, Malta; anthony-pio.dimech@gov.mt (A.P.D.); samuel-anthony.galea@gov.mt (S.A.G.); kevin.b.cassar@gov.mt (K.C.); 2 Faculty of Medicine and Surgery, University of Malta, MSD 2080 L-iMsida, Malta; 3 Department of Surgical Sciences, Section of Vascular Surgery, Uppsala University, 75185 Uppsala, Sweden

**Keywords:** peripheral arterial disease, diabetic foot, amputation, Malta

## Abstract

Introduction: Malta is a small island in the middle of the Mediterranean with a population of 514,564 inhabitants and is served by one public tertiary hospital, Mater Dei Hospital. The Vascular unit was set up in 2007. The aim of this review is to analyse the work related to peripheral arterial occlusive disease (PAOD) in Malta with an in-depth focus on amputations and revascularisation procedures since the introduction of the Vascular unit. Method: Various sources of data have been interrogated to address this subject. Population and prevalence data on obesity and type II diabetes mellitus from 2003 to 2019 was obtained from the National Statistics Office, the World Health Organization, and the International Diabetes Federation, respectively. The Maltese Vascular Register (MaltaVasc), and in-hospital reports from 2003 to 2019 was used to obtain data on revascularisation procedures, major amputations and minor amputation rates in Malta. Results: Malta has one of the highest rates of obesity in Europe. In 2015, the prevalence rate was 30.6%. Similarly, data from the International Diabetes Federation Atlas showed that the prevalence rate of T2DM among adults was 14% in 2017. There was a mean of 33 open/hybrid procedures per 100,000 population (28–38, 95% confidence interval) between 2005 and 2009 and a mean of 57 endovascular procedures per 100,000 population (46–68, 95% confidence interval) during the same time-period. From 2009 to 2019, there was a mean of 16 major amputations and 78 minor amputations per 100,000 population. Conclusion: A significant reduction in major amputation rates with an increase in minor amputation rates and revascularisation rates has been noted since the establishment of the vascular unit in Malta. During this period, there has been an increase in prevalence in obesity and T2DM together with an aging population.

## 1. Introduction

Malta is located about 90 km south of Sicily and consists of three inhabited islands, Malta, Gozo and Comino. Malta is the largest and most densely populated island with an area of 316 km^2^ while Gozo and Comino together cover 71.5 km^2^ [1]. The total population at the end of 2019 was 514,564 inhabitants with males being in the majority (265,762 (*n*); 51.6%). A total of 18% of inhabitants are older than 65 years, with 3049 of these aged 90 or over [1,2] (Table 1). Life expectancy is 82.5 years, which is slightly above the European average of 81 years [3].

The Maltese health service is largely provided by the state. Every Maltese citizen is covered by the Social Security Act and can access free health care provided by the state and funded through taxation. Primary health care services are provided by the state and private family medicine specialists (general practitioners, GPs). There is one main public teaching hospital in Malta, Mater Dei Hospital and one small public teaching hospital in Gozo. The vascular services are only present in Mater Dei Hospital and cover the Maltese Islands. A public rehabilitation hospital hosting an orthotics and prosthetics unit, a long-term care hospital and several homes for the elderly also form part of the public health service. The latter are supplemented by numerous private geriatric care homes. Patients with rare diseases or those requiring highly complex interventions are referred to centres abroad and funded by the state. The government supplies free medications for 85 chronic conditions listed under the second part of the Fifth Schedule of the Social Security Act (Schedule V) which include ischaemic heart disease, peripheral vascular disease and diabetes mellitus [1]. In 2017, health expenditure reached 9.3% of the GDP [5] with EUR 735 million budgeted for health in 2019 [6]. As of 2015, there were 391 physicians per 100,000 population in Malta, with 65% of these working in hospital [1].

## 2. Incidence and Prevalence of Atherosclerotic Diseases, PAOD, Diabetes Mellitus

**Figure 1:**
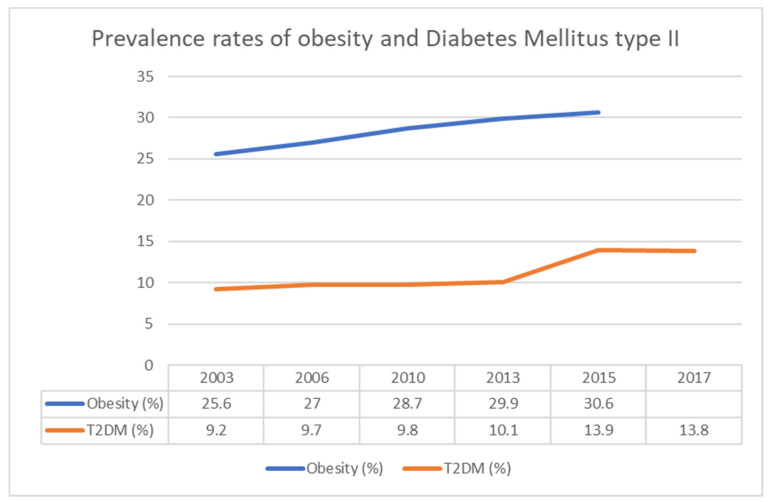
Percentage incidence of obesity and diabetes in the Maltese islands over the years.

According to global observatory data from the World Health Organization, Malta has one of the highest rates of obesity in Europe [7]. In 2003, this stood at 25.6% of the population but by 2015 it rose to 30.6% [7]. A total of 40% of Maltese school-aged children were overweight or obese in 2019 [8]. Similarly, data from the International Diabetes Federation Atlas shows that the prevalence rate of type II Diabetes Mellitus (T2DM) among adults aged between 20 and 79 years in Malta has increased steadily over the years: from 9% in 2003 to 14% in 2017 (Figure 1) [7]. Poor diet and blood glucose control, high smoking prevalence [9] and generalised lack of health awareness increase the population risk [7]. No national epidemiological data is available about the prevalence of atherosclerotic diseases, particularly PAOD.

## 3. Brief Description of How Higher Education, Training and Specialisation Is Organized from a Medical Student to the Vascular Specialist

Undergraduate medical training in Malta consists of a five-year programme at the University of Malta. For the first two years after graduation, doctors are employed with the National Health Service and enrolled in the Malta Foundation Programme, which is affiliated with the United Kingdom Foundation Programme. After successful completion of foundation training, doctors are eligible to apply for the position of Basic Specialist Trainee in several surgical specialties. Successful completion of basic specialist training entitles trainees to apply for a six-year higher specialist trainee post in vascular surgery. During this six-year programme, trainees are expected to gain additional experience in a foreign institution for at least one year and complete the final specialist exit examinations (European or British board exams) to become vascular specialists. After two years on the specialist register, they are eligible to apply for a consultant job when available [10].

## 4. Which Medical Specialties Are to What Degree Involved in PAOD Treatment? Who Performs Diabetic Foot Care and Amputations? How Is Multidisciplinary Team Decision Making Organised in Malta?

Specialist vascular services in Malta came into effect in 2007 with the appointment of the first consultant vascular surgeon. Before 2007, general surgeons with a vascular interest provided this care. Between 2008 and 2013, the workload was split between the vascular consultant and the aforementioned general surgeons whereby the vascular consultant was on-call every alternate week. From January 2014 onwards, all vascular cases fell under the care of the vascular team (two vascular consultants) with minimal involvement from general surgeons [11]. Over the years, the service expanded and, today, patients with PAOD in the Maltese islands are cared for by one of three consultant vascular surgeons. In addition, there are two recently appointed vascular resident specialists, five vascular higher specialist trainees, two basic specialist trainees and six foundation doctors. The latter two groups rotate between different specialities every three months. Together, these professionals form the vascular unit and provide emergency, urgent and elective vascular care on a 24/7 basis. During the on-call hours, there is a resident vascular specialist or a higher specialist trainee, with the consultant on-call being off-site. 

Referrals are received from all specialties, particularly general practice and the accident and emergency department. The vascular unit forms part of and manages multidisciplinary teams composed of interventional radiologists, microbiologists, podiatrists, physiotherapists, tissue viability nurses, a vascular practice theatre nurse. Weekly multidisciplinary meetings are held with microbiologists where inpatient antibiotic regimes are discussed and updated as necessary. A radiology multidisciplinary meeting is also organised every two weeks to discuss complex lower limb vascular pathologies amongst other pathologies. Regular joint clinics have been set up with dermatologists and tissue viability nurses dealing with complex lower limb ulceration. 

The vascular unit has a dedicated vascular podiatry clinic which is manned by a team of six podiatrists, two of whom are senior podiatrists. Apart from the planned follow up cases, the podiatrists also review patients with foot pathology on a walk-in basis. These podiatrists meet daily with the on-call vascular resident and patient reviews are conducted as needed. There is a separate weekly multidisciplinary outpatient diabetic foot clinic involving diabetologists, vascular surgeon and diabetic podiatrists. These clinics run in parallel with the weekly vascular surgery out-patients’ clinic held by each of the vascular consultants.

Patients with diabetic foot pathology requiring admission to hospital are admitted to a dedicated 15-bed Diabetic Foot Ward set up in 2018 with the primary aim to provide better holistic care to patients admitted with diabetic foot pathology. The lead physicians for diabetic foot pathologies are vascular surgeons but assisted by diabetologists, nephrologists, ophthalmologists and allied health professionals including but not limited to podiatrists, physiotherapists, orthotists and prosthetists. Patients with other vascular pathology are admitted to a dedicated 24-bed vascular ward. Post-operative and critically ill vascular patients are managed in the intensive care unit or cardiac intensive care unit or surgical high dependency unit, the latter two units serve as a vascular high dependency unit shared with cardio thoracic surgery and general surgery, respectively. 

A vascular ultrasound laboratory service within the vascular department is also available during working hours from Monday to Saturday. This is led by the vascular clinical lead and manned by four full-time vascular sonographers. The vascular sonographers provide urgent and elective duplex ultrasound. The vascular lab runs a vein bypass graft surveillance program whereby vein grafts are scanned prior to discharge, at 6 weeks, 3 months, 6 months, 12 months, 18 months, 24 months and then yearly. Significant graft stenoses are treated either via open or endovascular procedures. The vascular lab also provides an aneurysm surveillance programme in addition to carotid, venous and arterial scanning.

## 5. PAOD-Related Vascular Registry

The Malta Vascular Registry (MaltaVasc) consists of a database of patients who at some point came in contact with the vascular services on the island. Important details such as medical history, follow-up visits, imaging reports and interventions performed are all documented in this registry. In 2020, a short validation report was published in the European Journal of Vascular and Endovascular Surgery. When compared to official hospital administration records, the Malta Vascular Registry showed a 97% external validity and 100% internal validity for carotid and aortic aneurysm repairs performed between 2017 and 2018 [12]. Although not validated yet, the vascular registry also keeps records on open/hybrid procedures and endovascular (surgeons’ and intervention radiologists’) procedures for PAOD. Furthermore, two members of the vascular team are part of the ESVS VASCUNET committee and International Consortium of Vascular Registries (ICVR) and regularly take part in studies and sharing of outcomes as quality control.

## 6. How Are Patients Treated and Is There a Trend towards a Particular Technique?

**Figure 2:**
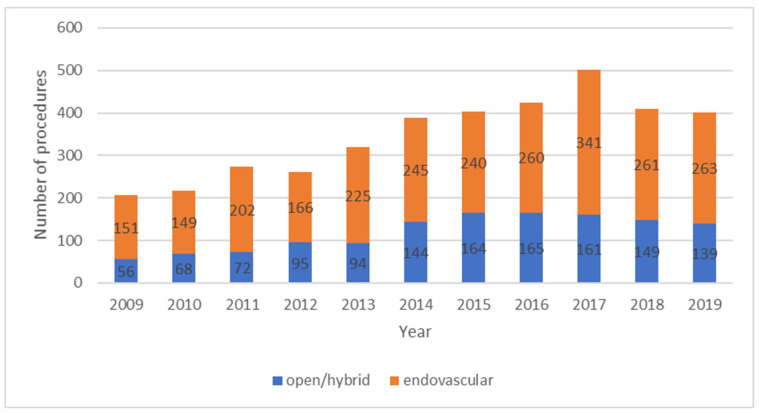
Number of vascular revascularization procedures carried out over the years by subtype.

Throughout the years, there has been a gradual increase in the number of revascularisation procedures. Through interrogation of MaltaVasc and population data provided by the National Statistics Office, open/hybrid revascularisation procedures have increased from 21 cases per year in 2003 to 139 cases per year in 2019, with a mean of 33 procedures per 100,000 population (28–38, 95% confidence interval) between 2005 and 2009. Using the ≥50 years of age population, there were 44 open/hybrid revascularisation per 100,000 people in 2011 vs. 75 open/hybrid revascularisation per 100,000 people in 2019, whilst no population data is available for pre-2011. Most of the revascularisation procedures are carried out by endovascular means (Figure 2). Purely lower limb endovascular procedures are carried out by interventional radiologists (IR) in a dedicated Angio suite. Along the years, the number of endovascular procedures carried out per year have increased and stabilised at a mean of 57 procedures per 100,000 population (46–68, 95% confidence interval) in the last five years (Figure 2). Open and/or hybrid surgery cases are performed in a dedicated vascular theatre by vascular surgeons; however, data is lacking on the number of purely hybrid procedures. The mean proportion of patients treated by endovascular procedures vs. open surgery between 2010 and 2017 was 68% (67–70, 95% confidence interval) in Malta [13]. This proportion was similar to that present in a number of European countries which participated in an international registry-based observation study [13]. Although Mater Dei hospital lacks a dedicated vascular hybrid theatre, plans to build one are ongoing and approval for this has already been obtained. Furthermore, all patients that require intervention are admitted to hospital and whenever possible, patients who receive endovascular care by the IR team are treated as day-cases.

## 7. Is There Any Development in Amputation Practice?

Minor and major amputations are primarily carried out by vascular surgeons. Only a handful per year are performed by other specialties such as orthopaedics and plastic surgery specialists. These are carried out exclusively for reasons other than vascular pathology such as due to malignancy and severe burn injuries.

**Figure 3:**
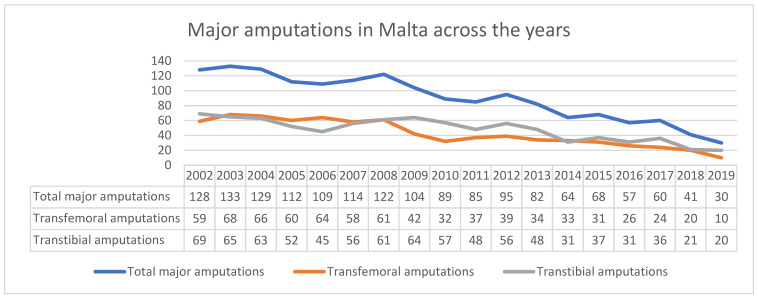
Number of major amputations over the years. Specialist vascular service was introduced in 2007 and became fully established in 2014.

**Figure 4:**
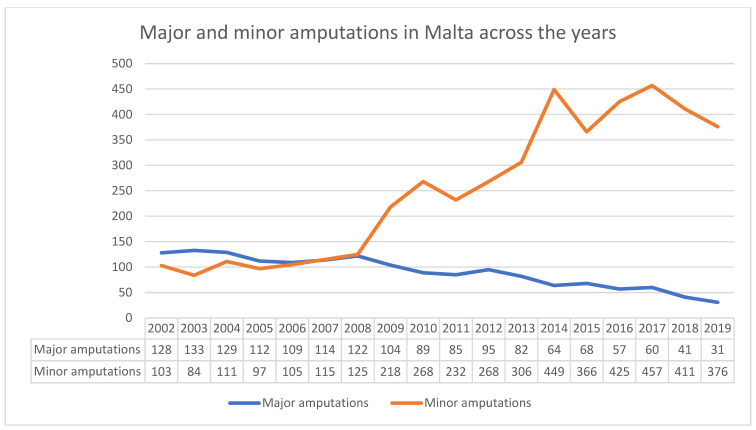
Comparison of major amputations (above ankle) with minor amputation rate (below ankle) between 2002 and 2019.

**Figure 5:**
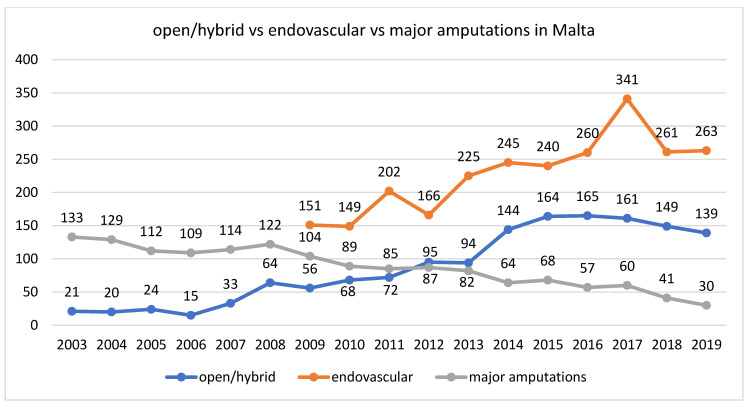
Comparison of open revascularization procedures with major amputation rate between 2003 and 2019.

Data from MaltaVasc and data from in-patient hospital reports show that after the introduction of specialist vascular service in Malta back in 2007, there was a steady decline in the yearly major amputation rate for peripheral arterial occlusive disease, from 109 in 2006 to 30 in 2019 (Figure 3). The number of major amputations carried out between 2010 and 2014 and the decline in major amputations rate compared well with most of the European countries that participated in the VASCUNET report [14]. In 2019, before the COVID-19 pandemic, only 6 per 100,000 population major amputations were carried out (Table 2). The reduction in major amputations not only leads to direct benefit to the patients but also leads to indirect benefit to community and health service [15]. On the other hand, minor amputation rates steadily increased during this time period, from an average of 100 per year (between 2002 and 2006) to 407 per year (between 2015 and 2019) (Figure 4), i.e., 229 minor amputations per 100,000 population for persons aged ≥ 50 years. Comparing the Maltese data to the VASCUNET group, more minor amputations were carried out than in any other European country which provided data to the VASCUNET report between 2010 and 2014 (Table 2). Although reasons for this exceptionally high rate of minor amputations are not known, potential reasons for this include the high prevalence rate of T2DM [7] coupled with a lack of a diabetic foot screening programme. Data from VASCUNET and ICVR show that the majority of revascularisation procedures in Malta are performed for chronic limb threatening ischaemia, as only 15% of revascularisation procedures are performed for intermittent claudication [13]. This further strengthens the belief that the large number of minor amputations are potentially due to high prevalence of T2DM and lack of a diabetic foot screening programme in the community. Although risk factors and other potential confounding factors have not been factored in, the data available is suggestive of an inverse relationship between minor and major amputation rates (Figure 4) and major amputation and revascularisation procedure rates (Figure 5). There is a similar relationship in Denmark; however, the positive correlation between revascularisation rate and amputation rate in Denmark was only significant between 2003 and 2008 [16]. Thus, further studies are required to examine the Maltese data in more detail and to determine whether the correlation between revascularisation and major amputation rate is significant.

Efforts are now being made to reduce the minor amputation rate. In order to address this issue, a dedicated vascular mobile phone line has been created so that allied health professionals and GPs can contact the vascular team directly on a 24/7 basis. Postgraduate lectures for doctors and nurses are being delivered as part of continued professional and practice development programs. Podiatrists are present in health clinics distributed around the country for regular diabetic foot reviews in the community and who, in turn, can contact the vascular surgeon on call and/or senior podiatrists at the main hospital as required. Discussions are also ongoing to create a registry of patients who suffer from diabetes mellitus to enable the setting up of a national diabetic foot screening programme. Furthermore, Public Health campaigns to address the risks factors for PAOD are currently in place. These include free smoking cessation classes/clinics and free healthy lifestyle (healthy eating and exercise) programs. All of the above will hopefully lead to a healthier population and reduce the risk of PAOD with all associated complications [17].

## 8. Conclusions

The vascular surgery unit in Malta is a relatively small and ‘new’ unit as part of the general surgery department at Mater Dei Hospital. Although the unit cares for a broad spectrum of vascular pathologies, the vast majority of the pathologies are related to PAOD due to the patients’ demographics. A significant reduction in major amputation rates with an increase in minor amputation rates and revascularisation rates has been noted since the establishment of the vascular unit. 

## Figures and Tables

**Table 1:** Population of the Maltese Islands by age group and sex as at 31 December 2019 [4].

Age Group	Males	Females	Total
0–9	24,486	22,786	47,272
10–19	22,700	21,332	44,032
20–29	42,896	36,082	78,978
30–39	48,450	40,451	88,901
40–49	37,503	32,552	70,055
50–59	30,635	28,533	59,168
60–69	29,646	29,905	59,551
70–79	21,139	23,779	44,918
80–89	7390	11,250	18,640
90+	917	2132	3049

**Table 2:** Number of major and minor amputations in Malta from 2009 to 2019.

Year	Total Population	Major Amputations (*n*)	Major Amputations per 100,000 Population	Minor Amputations (*n*)	Minor Amputations per 100,000 Population
2009	414,027	104	25	218	53
2010 *	414,989	89	21	268	65
2011 *	417,546	85	20	232	56
2012 *	421,364	95	23	268	64
2013 *	425,384	82	19	306	72
2014 *	429,344	64	15	449	105
2015	403,480	68	17	366	91
2016	440,433	57	13	425	96
2017	475,701	60	13	457	96
2018	493,559	41	8	411	83
2019	514,564	30	6	376	78

(* 2010–2014 data is provided for comparison with the VASCUNET report [14]).

## Data Availability

Data is freely available from cited documents.
